# Characteristic Fingerprint Based on Low Polar Constituents for Discrimination of *Wolfiporia extensa* according to Geographical Origin Using UV Spectroscopy and Chemometrics Methods

**DOI:** 10.1155/2014/519424

**Published:** 2014-12-02

**Authors:** Yan Li, Ji Zhang, Yanli Zhao, Zhimin Li, Tao Li, Yuanzhong Wang

**Affiliations:** ^1^Institute of Medicinal Plants, Yunnan Academy of Agricultural Sciences, Kunming 650200, China; ^2^College of Traditional Chinese Medicine, Yunnan University of Traditional Chinese Medicine, Kunming 650500, China; ^3^College of Resources and Environment, Yuxi Normal University, Yuxi 653100, China

## Abstract

The fungus species *Wolfiporia extensa* has a long history of medicinal usage and has also been commercially used to formulate nutraceuticals and functional foods in certain Asian countries. In the present study, a practical and promising method has been developed to discriminate the dried sclerotium of *W. extensa* collected from different geographical sites based on UV spectroscopy together with chemometrics methods. Characteristic fingerprint of low polar constituents of sample extracts that originated from chloroform has been obtained in the interval 250–400 nm. Chemometric pattern recognition methods such as partial least squares discriminant analysis (PLS-DA) and hierarchical cluster analysis (HCA) were applied to enhance the authenticity of discrimination of the specimens. The results showed that *W. extensa* samples were well classified according to their geographical origins. The proposed method can fully utilize diversified fingerprint characteristics of sclerotium of *W. extensa* and requires low-cost equipment and short-time analysis in comparison with other techniques. Meanwhile, this simple and efficient method may serve as a basis for the authentication of other medicinal fungi.

## 1. Introduction

For millennia, fungi have been appreciated by human beings as edible and medical resources. They are extraordinary species of natural medicines that have long been used around the world [1]. Medicinal fungi, which are used as decoctions and essences, are also normally applied as alternative medicine in China, Korea, Japan, and eastern Russia [2, 3]. Many species of fungi with pharmaceutical values are included in Chinese pharmacopoeia as traditional Chinese medicines (TCMs) [4].

Medicinal fungi showed their special therapeutic effects because of the complexity of their chemical components and different varieties of bioactivities [5]. However, chemical composition and bioefficacy are generally affected by the geographical origins, climatic conditions, environment, and other factors that may lead to somewhat different qualities of medicinal fungi even though they come from the same species [6, 7]. For example, the content of fatty acid in a very famous medicinal fungus,* Ophiocordyceps sinensis*, which should grow at least 3800 m above sea level [8], varied significantly due to difference in geographic origins [9]. When properly evaluating the health benefits of medicine chemical components in laboratory or clinical trials, one should know well where the raw material is from [10]. Therefore, a clear regional identity is imperative to guarantee quality and benefit for the exploitation and utilization of medicinal fungi. It was also the focus of attention for fungus growers.

In recent years, chemical fingerprints have attracted an increasing amount of interest and have been accepted as an available strategy for the identification and quality assessment of medicines by WHO and SFDA [11, 12]. Compared with conventional analytical approaches, fingerprint technique is focused on the holistic characterization of a complex system of a test sample [13, 14]. Numerous chemical fingerprint methods for discrimination and quality evaluation of medicinal fungi have been published including near infrared (NIR) spectroscopy, Fourier transform infrared (FT-IR) spectroscopy, high-performance liquid chromatography (HPLC), hydrophilic interaction chromatography (HILIC), gas chromatography-mass spectrometry (GC-MS), and DNA sequence analyses [15–20]. Moreover, these well-established analytical methods exhibited significant advantages for discrimination of different geographical origins when combined with chemometrics which focus on soft modeling for situations that are too complicated for the traditional hard models to work and get useful chemical information from the analytical data maximally [21–24]. However, these methods have a series of important drawbacks. For instance, FT-IR had the weaknesses that it needed the experienced technicians and it was hard to develop a suitable model [25]. For HPLC, the sample pretreatment used to be long and tedious, the standards and calibration are required, and it is a time-consuming, expensive, and destructive technique which is also difficult to implement in an online protocol [26]. Comparatively, UV spectra fingerprints which provide the comprehensive fuzz information of specimens and wildly used in medicine analysis have shown greater potential for discrimination of medicinal fungi [27–29]. Yang et al. used UV spectra fingerprints combined with multivariate analysis to discriminate boletes with different origins and species [30]. This approach is simple and cost-effective and could detect samples rapidly when coupled with chemometric data analysis techniques.

*Wolfiporia extensa* (Peck) Ginns, one species of wood-decaying fungi in the family Polyporaceae, is a well-known medicinal fungus widely used in China and certain Asian countries [31, 32]. This species gives large edible sclerotia which is one of the most important crude drugs normally used in the form of the decoctions and in combination with some other herbs in traditional Chinese and Japanese medicine [4, 33, 34].* W. extensa* contains two principal groups of chemicals: the triterpene fraction and the polysaccharide fraction [35]. Modern phytochemical and pharmacological researches demonstrated that main active constituents such as triterpenoids and polysaccharides isolated from* W. extensa* had antioxidant, antitumor, anticancer, anti-inflammatory, nematicidal activities, antihypertonic stress effect, and antihyperglycemic property [36–42]. This species fungus has not only long been utilized to treat a wide variety of diseases, but also recently has attracted the attention of the pharmaceutical industry. Traditionally, it has been used as a diuretic, sedative, and tonic to treat diabetes, edema, nephrosis, acute gastroenteric catarrh, chronic fatigue syndrome, insomnia, diarrhea, nausea, emesis, and dizziness [4, 31, 43]. What is more, it is commercially available and is popularly used in the formulation of nutraceuticals, cosmetics, tea supplements, and functional foods in Asia at present [44].

In this study, a practical and promising method has been developed to discriminate the dried sclerotium of* W. extensa* from different geographical origins based on UV spectroscopy. Low polar constituents of the specimens were analyzed by the established method. The spectroscopic data were analyzed by chemometric pattern recognition methods such as PLS-DA and HCA. This method could contribute to providing far more information for discriminating medicinal fungi rapidly and accurately.

## 2. Materials and Methods

### 2.1. Raw Materials

A total of 23 wild* W. extensa* sclerotium samples were obtained from different regions in Yunnan, southwestern China. Concretely, eight samples of Chuxiong, seven samples from Honghe, and eight samples collected in Pu'er were analyzed in this study. All the samples were authenticated by Dr. Honggao Liu from the College of Food Science and Technology, Yunnan Agricultural University, and preserved in the specimen room of Institute of Medicinal Plants, Yunnan Academy of Agricultural Sciences. The sample information is listed in Table 1.

### 2.2. Sample Preparation

All the fresh samples were cleaned up and air-dried in the shade after collection. Then they were ground into powder and passed through a 100-mesh stainless steel sieve. The sieved powders were stored in the labeled Ziploc bags at room temperature until further analysis. Each sample (1.00 g) was dissolved in 10.0 mL of chloroform (analytical grade), which provided a solvent of low polar constituents of* W. extensa* and extracted by ultrasonication for 40 min. The extracts then were filtered and kept as stock solutions for testing.

### 2.3. Ultraviolet Spectroscopy

Each stock solution was analyzed by UV spectroscopy using a TU-1901 PC UV-visible spectrophotometer (Pgeneral, Beijing, China) equipped with a quartz cell with an optical path of 1 cm. The absorption spectra were collected in the working range from 190 to 450 nm with 0.5 nm sampling interval and 2.0 nm slit width. Then the raw spectra were treated by smoothing in order to eliminate the solvent interference and increase accuracy of spectra. The obtained absorption readings of all the samples were converted into a data matrix by using Microsoft Excel 2007 (Microsoft, USA) with the wavelength as variables represented by columns and the corresponding spectral absorbance measurements of different samples represented by rows.

### 2.4. Data Analysis

In order to sort the* W. extensa* sclerotium samples according to their geographical origins, the spectrum data matrices were integrated and exported to the appropriate software for chemometrics analysis of the spectra. The chemometric techniques such as multivariate classification methods aimed at finding mathematical models were able to recognize the membership of each sample to its appropriate class, on the basis of a set of measurements [45]. In this study, partial least squares discriminant analysis (PLS-DA) and hierarchical cluster analysis (HCA) were used as multivariate tools. The SIMCA-P^+^ 10.0 (Umetrics, Umeå, Sweden) was used for the PLS-DA modeling, while HCA was carried out using SPSS 20.0 (IBM Corp., Armonk, USA).

According to the algorithm of PLS-DA [46], the spectra of training set can be represented as an *n* × *p* matrix *X*, where *n* means training objects and *p* stands for wavelength points. Then an *n* × *s* matrix *Y* is designed. *s* is the value of sorted number in this study, *s* = 3 (the classes of Chuxiong, Honghe, and Pu'er, resp.). The value of each element in *Y* is the corresponding class of the object in *X*. If an object *i* (*i* = 1 : *n*) is from class *j* (*j* = 1 : *s*), the element at *i*th row and *j*th column in *Y* is given a value of 1. All other elements in *Y* are set as −1. PLS-DA was selected to obtain the first understanding of the relationships among the data matrix and employed to distinguish samples according to their origins. Then, for HCA, the squared Euclidean distance and the average linkage method were used. The main principle of HCA is assuming that there are *m* observations; then the algorithm starts with *m* clusters. With the calculation of the squared Euclidean distance between observations, the closest points are grouped into a single cluster and repeat the process until all the observations are included in one cluster [47]. This method was utilized to evaluate the relationships in terms of similarity or dissimilarity among groups of multivariate data.

## 3. Results and Discussion

### 3.1. Optimization of Extraction Methods

**Figure 1:**
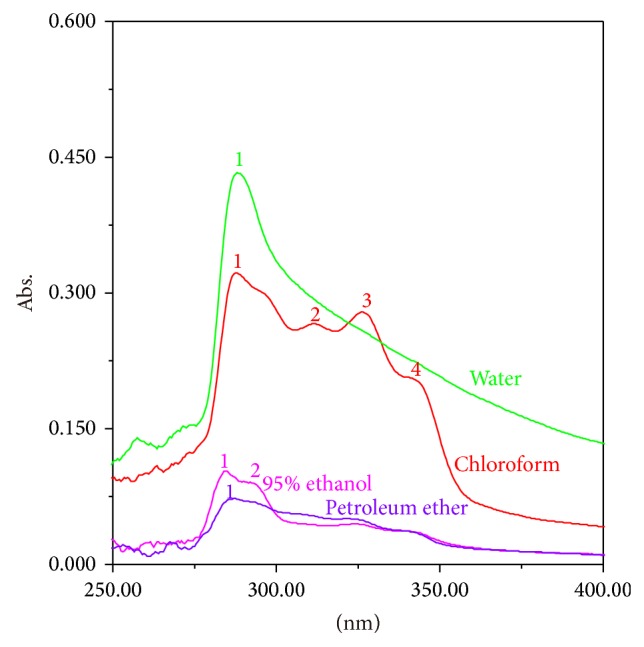
UV spectra of different extraction solvent.

**Figure 2:**
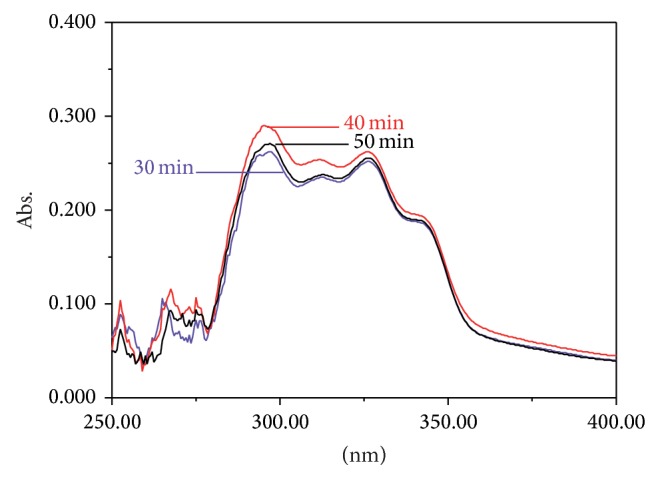
UV spectra of different extraction times.

Six hundred milligrams of every powdered sample was taken out to form the mixed* W. extensa* sclerotium sample. The mixed sample was used to make sure of the optimization of extraction methods. Efficient extraction methods are also required for the highest extraction efficiency [48]. In order to obtain the efficient extraction methods, the extraction solvent and ultrasonic time tests were investigated. The number of the absorption peaks from four different extracts which were extracted by petroleum ether, chloroform, 95% ethanol, and ultrapure water was used to validate the extraction solvent while different extraction times (30, 40, and 50 min) were screened based on the intensities of absorption bands. All reagents were of analytical grade. The results showed that chloroform could be the most appropriate solvent.  Figure 1 shows that the number of the absorption peaks of the chloroform extract is the highest among all the extracts. Others have only one or two absorption peaks. This implied that chloroform extract may obtain more component information about the sample to reflect its characteristic. Moreover, all spectrophotometric signals were maximized with 40 min of extraction and a longer time was not necessary (Figure 2).

### 3.2. Validation of Methodology

To ensure the validity of this proposed method, the method precision was performed on seven replicate determinations of the extract of mixed sample with the selected condition. The variation of wavelength of common peaks was expressed as relative standard deviation (RSD). The RSDs of precision for this method were less than 1.08%. The repeatability was assessed by testing seven independently prepared extracts which were from the mixed sample using the uniform method. The RSDs of wavelength of common peaks were arranged from 0 to 0.58%. The sample stability was determined by analysing a single sample solution stored at room temperature for 30 h. The RSDs from stability test were below 1.02% for all the wavelengths of common peaks, indicating that* W. extensa* sclerotium extraction solution was stable within 30 h. These results displayed that this method was reasonable.

### 3.3. Spectroscopic Analysis of Low Polar Constituents of* W. extensa* Sclerotium

**Figure 3:**
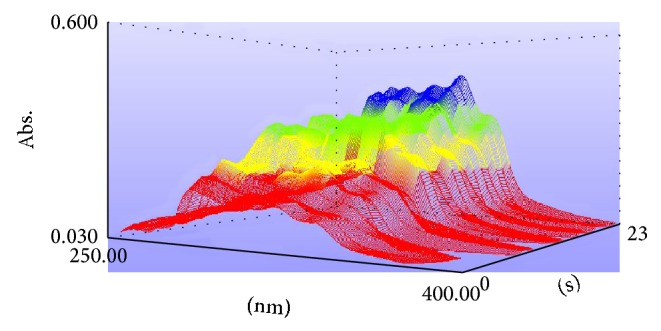
Three-dimensional wireframe plot of UV spectra of* W. extensa* sclerotium specimens.

**Figure 4:**
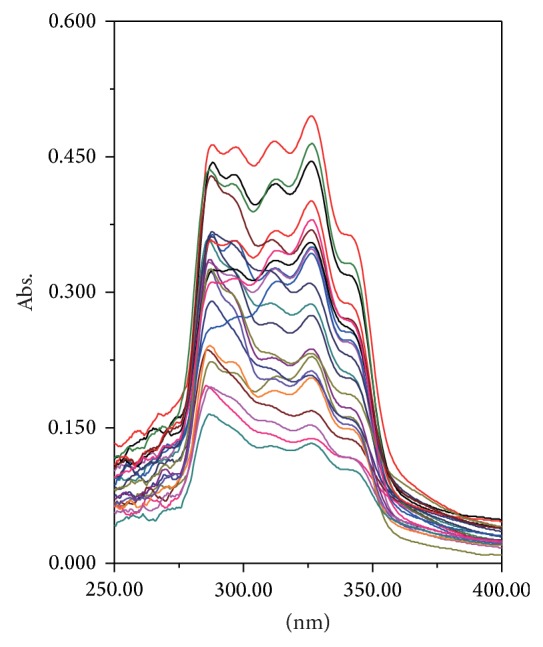
Two-dimensional spectra diagram of* W. extensa* sclerotium samples.

The UV absorption bands of the presented samples are usually associated with the presence of different chromophores exemplified in conjugated systems as well as other UV-absorbing systems [49]. The UV spectrum of each of the studied* W. extensa* sclerotium samples was recorded in the region between 190 and 400 nm. On account of the detection range of the UV-visible spectrometer, we chose the wavelengths of absorption peaks arranged from 250 to 400 nm for the sake of avoiding the spectral noise. The UV spectra for all the samples are presented in Figures 3 and 4.

In the three-dimensional wireframe plot of UV spectra (Figure 3), the red part means the absorbance is recorded in the region between 0 and 0.2, the yellow one means the absorbance is arranged from 0.2 to 0.3, for the green part, the absorbance is recorded from 0.3 to 0.4, and the blue one which means the absorbance is relatively high is arranged from 0.4 to 0.5. Just a few samples have higher absorbance, which have been shown in blue. It indicated that the absorbance of low polar constituents of different samples has visible differences. To a certain degree, when the substance was in high concentration, the corresponding absorbance was high too [50]. It implied that the contents of low polar constituents of the samples may differ with the geographical origins. For the two-dimensional spectra diagram (Figure 4), the UV spectra fingerprints of low polar constituents of* W. extensa* sclerotium have high overlap rate from 250 to 400 nm. Every sample has some characteristic absorption peaks to show its fingerprint feature. Some low polar constituents appear to be very similar among these samples because all the samples have some common peaks such as 287, 312, 326, and 340 nm. However, there were obviously differences among the number of absorption peaks and peak positions of these samples. These differences were conducive to discriminate the* W. extensa* sclerotium samples by showing the fingerprint characteristics.

### 3.4. Partial Least Squares Discriminant Analysis

**Figure 5:**
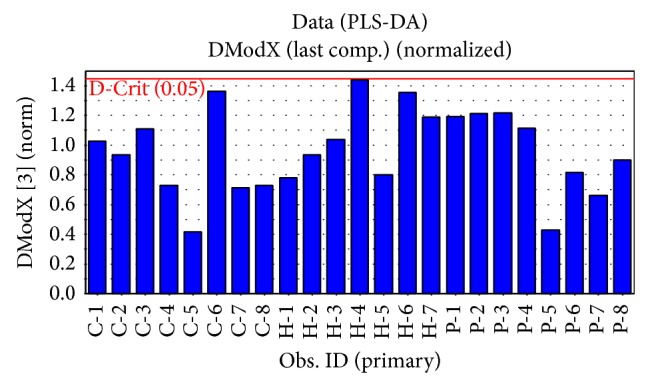
Distance to model in X-space (DModX) of all the specimens.

PLS-DA, a supervised method, is a variation of PLS analysis. It is considered as a pair comparison analysis and is built to classify a group of samples as belonging or not belonging to a specific class [51, 52]. This method, as a representative technique, was applied to construct and validate a statistical model to find difference in low polar constituents among the* W. extensa* sclerotium samples according to their geographical origins.  Figure 5 reports the distance to model in X-space (DModX) of all the samples. The values of DModX of all samples are under 1.45 and a value of *P* < 0.05 is considered statistically significant. It revealed that the results of PLS-DA were reasonable.

**Figure 6:**
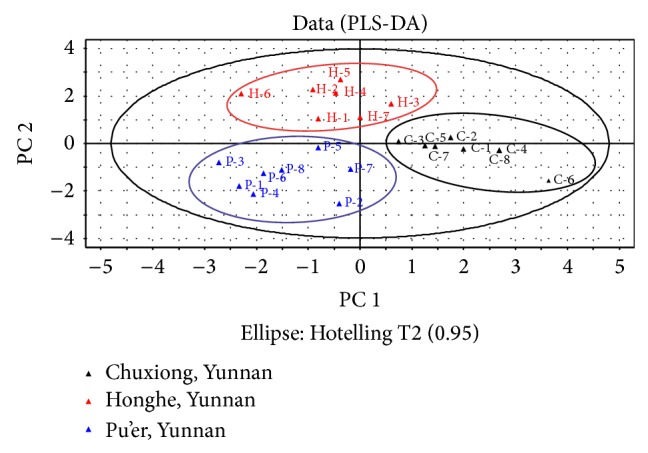
PLS-DA score plot based on UV spectra of* W. extensa* samples.

Figure 6 presents a score plot with 95% confidence ellipses obtained by applying PLS-DA to the overall set of UV spectra. A separation among the sclerotium samples which were clustered into three classes according to samples collected from Chuxiong, Honghe, and Pu'er, respectively, can be clearly observed in the two-dimensional diagram. As can be seen, it demonstrated the absence of significant variance within the same variety that the samples which were collected from the same origin could get together with each other and be distinct from others. This finding indicated that the spectral differences among these samples were systematic and can be used for discrimination purposes. Moreover, it could give us a preliminary overview of similarities and diversity among the geographical origins. In addition, all the sample symbols are in the ellipse that proved the effectiveness of the PLS-DA score plot as a convenient visualization technique for the differentiation. As a result, 23 test samples were discriminated entirely as their groups by geographical origins.

**Figure 7:**
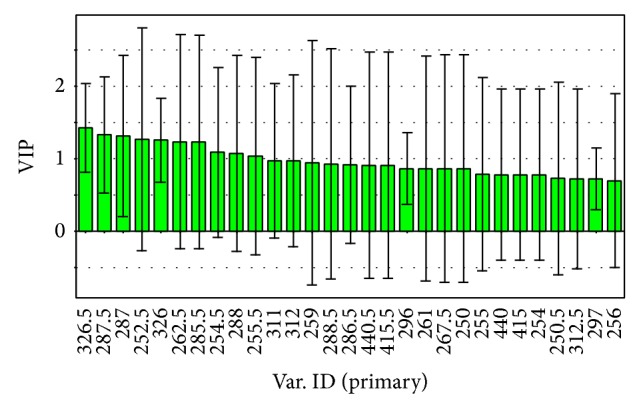
Variable importance for the projection (VIP) plot of absorbance for the contribution to sample separation from PLS-DA.

**Figure 8:**
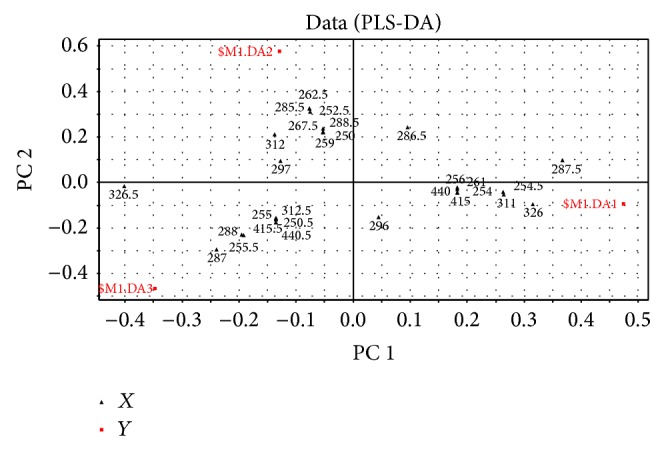
Loading plot generated from the PLS-DA model of the* W. extensa* samples.

According to the spectrophotometric PLS-DA model, a series of scores (variable importance for the projections (VIPs)) were computed to express the contribution of absorbance to these dimensions. A variable is usually considered important to the model when its VIP is above 1.0 [53].  Figure 7 and Table 2 have shown the VIP scores of the PLS-DA. The samples' data are easily discernible. Components that play important roles in the separation are picked out according to the parameter VIP. As shown in this figure, the absorption of the wavelength of 326.5, 287.5, 287, 262.5, 285.5, 326, 252.5, 255.5, 288, 311, 254.5, and 312 nm is likely to be considered as main factor for discrimination of all these specimens. From the corresponding loading plot (Figure 8), PC 1 resolves the measured composition profiles of specimens collected from Chuxiong from other samples. This variation was mainly attributable to the absorbance of wavelength of 326, 311, and 254.5 nm, of which the loading values were positive. In addition, the samples of Honghe were separated from those of Pu'er by PC 2 in which the corresponding loading values were positive for the absorbance of wavelength of 312, 285.5, 262.5, and 252.5 nm. What is more, these methods could screen suitable wavelengths to provide references for the quantitative test. Combining the spectra diagram with the VIP scores, the wavelengths of 287 and 326 nm may be appropriate for quantitative test because they were the common peaks of the samples and had the VIPs greater than 1.0 as well.

### 3.5. Hierarchical Cluster Analysis

**Figure 9:**
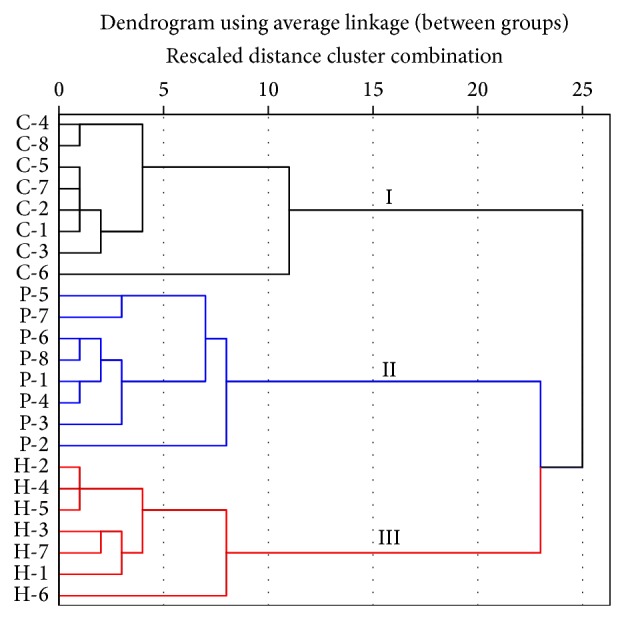
Dendrogram resulting from hierarchical cluster analysis.

HCA is an unsupervised pattern recognition method for clustering samples based on their similarities [54, 55]. To further explore the relationships among the* W. extensa* sclerotium specimens, HCA of the spectra data was performed.  Table 3 is the agglomeration schedule that shows the detailed steps of HCA. The corresponding cluster dendrogram was generated by applying hclust function using average linkage clustering of the squared Euclidean distance based on the normalized data from the 23 test samples. As shown in Figure 9, all the specimens could be divided into three fractions when the distance of them is twenty, group I contains the samples of Chuxiong, and the other two groups are composed of the samples collected from Pu'er and Honghe, respectively. All samples were correctly classified according to their geographical origins without any misclassification. In addition, the results could verify the consequence of PLS-DA.

It suggested that the low polar constituents in* W. extensa* sclerotium collected from the same area may be similar while the accumulation of these chemical constituents in samples that had different collection sites was likely to be diverse. This may be related to the local environment factors such as temperature, rainfall, soil type, vegetation type, or other characteristics. Similar results have been reported in the previous researches that phytochemical composition and metabolites of medicinal fungi could be affected by the geographical origins [9, 56]. This study implied that samples collected from different sites could be clearly distinguished by UV characteristic fingerprint based on low polar constituents when combined with chemometrics. Compared with the similar study that uses UV spectra fingerprint in combination with the common and variation peak ratio dual index sequence analysis for qualitative evaluation and reveals the differences of specimens collected from different areas [57], our study has the advantageous aspects that the results were visualized for the differentiation of samples and it was a convenient approach avoiding the tedious calculation. In addition, in contrast to the results of previous studies related to the discrimination of medicinal fungi based on other analytical approaches, such as HPLC [17] and GC-MS [58], it can be concluded that the proposed method is a reliable and fast tool for discriminating medicinal fungi.

## 4. Conclusions

In conclusion, a novel, fast, and convenient method has been developed to differentiate the sclerotium of* W. extensa* from different geographical sites based on low polar constituents by using UV spectroscopy coupled with chemometrics methods. All the samples could be discriminated accurately according to their origins. Even though the proposed method is qualitative, it avoids the need of a quantitative method that would require the use of standards, calibration, and time-consuming analysis. Furthermore, this approach is simple, of low cost, and reliable and has a significant advantage for discrimination of other species of medicinal fungi.

## Figures and Tables

**Table 1:** Information of all the wild *Wolfiporia extensa *(Peck) Ginns samples.

Code	Geographical origin
C-1	Chuxiong, Yunnan
C-2	Chuxiong, Yunnan
C-3	Chuxiong, Yunnan
C-4	Chuxiong, Yunnan
C-5	Chuxiong, Yunnan
C-6	Chuxiong, Yunnan
C-7	Chuxiong, Yunnan
C-8	Chuxiong, Yunnan
H-1	Honghe, Yunnan
H-2	Honghe, Yunnan
H-3	Honghe, Yunnan
H-4	Honghe, Yunnan
H-5	Honghe, Yunnan
H-6	Honghe, Yunnan
H-7	Honghe, Yunnan
P-1	Pu'er, Yunnan
P-2	Pu'er, Yunnan
P-3	Pu'er, Yunnan
P-4	Pu'er, Yunnan
P-5	Pu'er, Yunnan
P-6	Pu'er, Yunnan
P-7	Pu'er, Yunnan
P-8	Pu'er, Yunnan

**Table 2:** VIP scores of PLS-DA.

Var. ID (primary)	VIP
326.5	1.51178
287.5	1.40302
287	1.38853
262.5	1.30931
285.5	1.30725
326	1.27077
252.5	1.25123
255.5	1.10071
288	1.09983
311	1.0315
254.5	1.01802
312	1.00679
286.5	0.971985
288.5	0.953564
267.5	0.901482
250	0.901482
259	0.88801
440.5	0.810888
415.5	0.810888
250.5	0.773825
312.5	0.767684
255	0.759275
440	0.720724
415	0.720724
254	0.720724
256	0.707384
261	0.699291
297	0.627387
296	0.62471

**Table 3:** Agglomeration schedule of HCA.

Stage	Cluster combination		Coefficients	Stage cluster first appearance		Next stage
	Cluster 1	Cluster 2		Cluster 1	Cluster 2	
1	4	8	0.000	0	0	15
2	5	7	0.040	0	0	6
3	21	23	0.142	0	0	11
4	16	19	0.187	0	0	11
5	10	12	0.216	0	0	8
6	2	5	0.292	0	2	7
7	1	2	0.393	0	6	10
8	10	13	0.395	5	0	16
9	11	15	0.659	0	0	14
10	1	3	0.881	7	0	15
11	16	21	0.947	4	3	13
12	20	22	1.196	0	0	17
13	16	18	1.457	11	0	17
14	9	11	1.526	0	9	16
15	1	4	1.834	10	1	20
16	9	10	1.984	14	8	19
17	16	20	3.922	13	12	18
18	16	17	4.365	17	0	21
19	9	14	4.569	16	0	21
20	1	6	6.022	15	0	22
21	9	16	13.409	19	18	22
22	1	9	14.743	20	21	0
